# Material Properties and Constitutive Modeling of Infant Porcine Cerebellum Tissue in Tension at High Strain Rate

**DOI:** 10.1371/journal.pone.0123506

**Published:** 2015-04-01

**Authors:** Kui Li, Hui Zhao, Wenjun Liu, Zhiyong Yin

**Affiliations:** Institute for Traffic Medicine, Department 4th, Institute of Surgery Research, Daping Hospital, Third Military Medical University, Chongqing, 400042, China; Brandeis University, UNITED STATES

## Abstract

**Background:**

The mechanical characterization of infant porcine cerebellum tissue in tension at high strain rate is crucial for modeling traumatic cerebellum injury, which is in turn helpful for understanding the biomechanics of such injuries suffered in traffic accidents.

**Material and Method:**

In this study, the infant porcine cerebellum tissue was given three loading velocities, ie, 2s^-1^, 20s^-1^ and 100s^-1^ with up to 30% strain to investigate the tensile properties. At least six tensile tests for each strain rate were validly performed. Fung, Gent, Ogden and exponential models were applied to fit the constitutive equations, so as to obtain material parameters from the experimental data.

**Results:**

The Lagrange stress of infant porcine cerebellum tissue in tension appeared to be no more than 3000Pa at each loading velocity. More specifically, the Lagrange stress at 30% strain was (393.7±84.4)Pa, (928.3±56.3)Pa and (2582.4±282.2)Pa at strain rates of 2s^-1^, 20s^-1^ and 100s^-1^, respectively. Fung (0.833≤R^2^≤0.924), Gent (0.797≤R^2^≤0.875), Ogden (0.859≤R^2^≤0.944) and exponential (0.930≤R^2^≤0.972) models provided excellent fitting to experimental data up to 30% strain.

**Conclusions:**

The infant cerebellum tissue shows a stiffer response with increase of the loading speed, indicating a strong strain-rate sensitivity. This study will enrich the knowledge on the material properties of infant brain tissue, which may augment the biofidelity of finite element model of human pediatric cerebellum.

## Introduction

High quality experimental data on the mechanical characterization of infant human cerebellum tissue is essential for enhancing the bio-fidelity of computational pediatric cerebellum models, such as finite element pediatric cerebellum models, which can accurately simulate the brain’s response to complex loading conditions. However, due to ethical problems and difficulty in obtaining human cerebellum tissue, the infant porcine tissue may serve as a good substitute [1, 2]. To date, the mechanical property of living tissue is one of the central parts of biomechanics [3]. A wealth of literatures is available about the mechanical properties of cerebrum tissue for dynamic oscillatory shear tests [4, 5] and unconfined compression tests [6, 7, 8]. However, the properties of cerebrum tissue in tension are less well characterized than those in other loading modes, with only a few studies reporting tensile properties [9, 10, 11]. The possible main causes are the technical problem of conducting extension tests and the lack of an analytical relation between the tensile head displacement and strain for cylindrical samples with low aspect ratios [12]. Previous studies have investigated the effect of different loading rates, breeding, gender, temperature, friction, region, direction, age, and sample preparation, but some experimental results showed no consistent similarities from each other [13, 14]. However, it is generally understood that cerebrum tissue is sensitive to strain rate, even at very high loading rates of 1000–3000 s^-1^ [1].

Most previous studies about the mechanical properties of cerebrum tissue have focused exclusively on adult cerebrum tissue, whereas few attentions were paid to the cerebellum, despite its importance in the control of motion and the clinical prevalence of cerebellar pathologies. Due to the absence of cerebellum mechanical properties data, the similar mechanical properties to the remainder of the brain were implemented into the cerebellum in the current models of brain trauma [15]. Zhang et al [15] characterized the viscoelastic properties of living human cerebellum using magnetic resonance elastography, and found that the cerebellum may respond differently to the mechanical loading than the cerebrum. Christ et al [16] reported mechanical differences between white and gray matter in rat cerebellum by using scanning force microscopy. In addition, Prange and Margulies have investigated the age-dependency of cerebrum material properties and found significant difference between infant and adult porcine tissue [2]. There are little reports concerning the infant cerebellum tissue in tension at high strain rate. Thus, it is necessary to describe the mechanical behavior of infant cerebellum tissue.

In order to give more insight into the brain tissue behavior under surgical condition, Miller and Chinzei performed compressive and tensile tests on brain tissue at considerably lower strain rate of 0.000064, 0.000064, and 0.64s^-1^ [3, 9]. Similarly, for the purpose of updating the mechanical properties of brain tissue under traumatic events, Tamura et al [10, 17]conducted compression tests at strain rate of 1 s^-1^, 10 s^-1^, and 50s^-1^, while 0.9 s^-1^, 4.3 s^-1^, and 25s^-1^ in the tension tests. Rashid et al [8, 11] conducted the compression and tension tests to 30% strain at strain rate of 30s^-1^, 60s^-1^, and 90s^-1^. The review of previous literatures suggested that the strain associated with traumatic brain injury threshold is greater than 0.2 applied at strain rate in the range of 1-100s^-1^ [10, 11, 18]. Whereas peak strains up to 30%-50% were suggested for the onset of failure in compression [1]. In this study, the mechanical properties of infant porcine cerebellum tissue were determined by performing a set of uniaxial tension tests to 30% strain at different loading velocities of 2s^-1^, 20s^-1^, and 100s^-1^ that are of direct relevance to traumatic brain injury so as to better understand the mechanical property of infant cerebellum.

## Materials and Methods

Ethical approval for this research was obtained from the Research Ethics Committee of the Third Affiliated Hospital of Third Military Medical University, and this study was carried out in strict accordance with the recommendations in the guide for the care and use of laboratory animals of the National Institutes of Health. All efforts were made to minimize suffering of the animals.

### Specimen preparation

**Fig 1:**
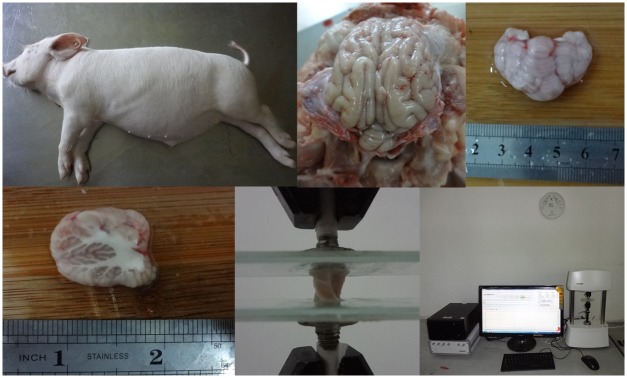
Specimen preparation and test apparatus for high-rate extension. A. Infant swine. B. Fresh brain tissue after removing dura matter. C. Infant cerebellum tissue. D. Infant cerebellar hemispheres. E. Porcine brain tissue sample subjected to extension. F. The biomaterials test instrument at the consistent room temperature (about 22°C).

As shown in Fig. 1, a total of 23 infant pigs, aged four weeks and weighting (16±0.5) kg were involved in this study and the cerebellum tissues were harvested immediately after the pigs were killed as a by-product at a local slaughter house in Chongqing BORN Biological Technology Co., Ltd., Chongqing, and all the tests were finished within 6h postmortem. After being removed from the dura, each cerebellum was preserved in a physiological saline solution (0.9% NaCl, 154mmolL^-1^) held in a thermal insulation stainless vessel at 4°C–5°C during the transportation. The specimens should be avoided being frozen at any time during the procedure. The cerebellums were removed from the brain stem by incising through the middle cerebellar peduncles, and the cerebellum tissue was split into right and left halves by cutting through the sagittal midline of the cerebellar vermis. One half of the cerebellar hemisphere was cut in the sagittal plane to extract one sagittal slice. Cylindrical specimens (9 mm in diameter and 5 mm in height) were slowly cored out from the center of the cerebellar slice using a steel circular cylindrical trephine (9 mm in diameter) with sharp edges. The faces of the cylindrical cerebrum specimens were then smoothed manually using a surgical scalpel. These samples consisted of approximately equal amounts of white and gray matter. The actual diameter and thickness of the specimen under stress-free conditions were (9.0±0.1)mm and (5.0±0.2)mm, measured with a digital slide caliper. Physiological saline solution was applied frequently to the specimens during cutting and before testing for preventing dehydration. Another sample would not be acquired from the cerebellar hemisphere until the test for previous sample was finished.

### Experimental setup

The tensile testing was performed using a computer-controlled, high-precision testing device adapted for biological specimens (Bose ElectroForce 3100, Bose Corporation, Gillingham, UK, Fig. 1). The major components of the testing rig include an electromagnetic driven motor, with a stroke resolution of 0.0015mm, a maximum stroke length of 5mm, and a minimum load resolution of 6mN with a 22N load cell. For the present work, this biomaterials test instrument enabled us to perform tensile tests to characterize the behavior of infant cerebellum under traumatic events. The displacement (mm) and force (N) data were recorded against time(s) for the tissue while experiencing 30% strain. It could be assumed that the machine had a quick response to achieved instantly the prescribed loading speed.

### Experimental protocol

As indicated by Miller and Chinzei [9], one challenging issue is how to place the samples in the testing machine in a reliable and repeatable way. In the present study, all the experiments were tested at room temperature (about 22°C). Cylindrical tissue samples were axially extended between two glass slides, which were rigidly fixed to the testing apparatus by tensile grips and clips. The surface of the glass slides was first covered with a thin layer of surgical glue (Gyanoacrylate, low-viscosity Z105880-1EA, Sigma-Aldrich), and then the prepared cylindrical tissue specimen was placed on the lower glue-coved glass slide. After fixing the bottom surface of the specimen, the upper glass slide with a thin layer of the surgical glue was lowered down slowly to touch the top surface of the specimen. To achieve excellent adhesion of the tissue to the upper and lower glass slides, the moving was stopped when the load was up to 5mN, and the upper glass slide was maintained in the position for one minute. As a result, the bottom and top surfaces of the tissue were rigidly attached to the moving and supporting glass slides. The velocity of the moving slide was adjusted to 10mm/s, 100mm/s, and 500mm/s to attain strain rates of 30s^-1^, 60s^-1^, and 90s^-1^, respectively. Each sample only executed one loading cycle. No preconditioning was performed because of the extreme delicacy and tackiness of the cerebellum tissue. Care was taken to observe whether the upper or lower faces of the specimen remained adhered to the moving and supporting glass slides during the extension phase. The data were not enrolled in the latter analysis if the ends of the specimen were not firmly bonded to the moving and supporting slides during extension of the cerebellum specimen.

### Data processing and analysis

To data, there is no widely accepted constitutive model for brain tissue that is able to match the full spectrum of the strongly strain rate sensitive, non-linearly viscoelastic behavior of the brain tissue [1]. According to the recorded force and distance, the constitutive models of cerebellum tissue were developed based on the typically used hyperelastic models, in which a strain energy potential function deriving the relationship between stress and strain tensors was used. The strain energy function, *W*, was usually defined in terms of the invariants (*I*_*1*_, *I*_*2*_, *I*_*3*_) of the strain tensor. As an incompressible material, the third strain (*I*_*3*_) is unity. Hence the strain energy function is a function of the first two invariants only: *W = W* (*I*_*1*_, *I*_*2*_), where *I*_*1*_ and *I*_*2*_ are defined as:

$$I_{1}\;=\;\lambda_{1}^{2}+\lambda_{2}^{2}+\lambda_{3}^{2}$$ (1)

$$I_{2}\;=\;\lambda_{1}^{2}\lambda_{2}^{2}+\lambda_{1}^{2}\lambda_{3}^{2}+\lambda_{2}^{2}\lambda_{3}^{2}$$ (2)

Where $\lambda_{1}^{2}$,$\lambda_{2}^{2}$, and $\lambda_{3}^{2}$ are the squares of the principal stretch ratios [19]. In this study, the homogenous deformation of specimen was achieved based on approximately uniform contraction of the cerebellum specimen during the extension phase. Then, the Eulerian and Lagrangian principal axes of strain and stress are aligned with direction of tension, and with any two orthogonal axes (lateral). Due to symmetry and incompressibility, the stretch ratios are indicated as the following form:

$$\lambda_{1}\lambda_{2}\lambda_{3}\;=\;1$$ (3)

$$\lambda_{1}\;=\;\lambda \;and\;\lambda_{2}\;=\;\lambda_{3}\;=\;\frac{1}{\sqrt{\lambda }}$$ (4)

Where λ is the stretch ratio in the direction of tension, then the strain energy function may be descripted as the below form:

$$W\;=\;W(\lambda^{2}+2\lambda^{-1},\lambda^{-2}+2\lambda )$$ (5)

From the transformation, the strain energy function is only a function of λ. For the extension test with confined conditions, the stretch ratio λ is calculated from the measure of the elongation *e* using equation: λ = 1+*e*, which is proportional to the change in total height if the change $\frac{h}{H}$ is within 1.0~1.3 [12], when value of λ was calculated by the bellow form:

$$\lambda \;=\;K\left(\frac{h}{H}-1\right)+1,\;K\;=\;1.583$$ (6)

And the Lagrange stress component along the direction of tension S_11_ was determined as:

$$S_{11}\;=\;\frac{F}{A}$$ (7)

Where F is measured tension force in Newton from the load cell of the machine, and A is the area of a cross section for the specimen. Then, the experimentally measured Lagrange stress was compared to the predicted value from the hyperelastic models from the below relation:

$$S_{11}\;=\;\frac{d\tilde{w}}{d\lambda }$$ (8)

Finally, the material parameters of some common hyperelastic models were adjusted to obtain good curve fitting. The chosen common hyperelastic models included the strain energy function of Fung, Gent, and Ogden, as illustrated in the following form. The Fung strain energy function was often used for the modeling of soft biological tissues in tension [20, 21].

$$W\;=\;\frac{\mu }{2b}\left[e^{b\left(I_{1}-3\right)}-1\right]$$ (9)

$$S_{11}\;=\;{\mu e}^{b\left(\lambda^{2}+2\lambda^{-1}-3\right)}\left(\lambda -\lambda^{-2}\right)$$ (10)

Gent strain energy function was capable to describe strain-stiffening material in a very satisfying way [22].

$$W\;=\;-\frac{\mu }{2}J_{m}\ln\left(1-\frac{I_{1}-3}{J_{m}}\right)$$ (11)

$$S_{11}\;=\;\frac{\mu J_{m}}{J_{m}-\lambda^{2}-2\lambda^{-1}+3}\left(\lambda -\lambda^{-2}\right)$$ (12)

Ogden strain energy function has been proved fully suitable for characterizing the cerebrum tissue in previous studies [11, 23, 24].

$$W\;=\;\frac{2\mu }{\alpha^{2}}\left(\lambda_{1}^{\alpha }+\lambda_{2}^{\alpha }+\lambda_{3}^{\alpha }-3\right)$$ (13)

$$S_{11}\;=\;\frac{2\mu }{\alpha }\left\{\lambda^{\alpha -1}-\lambda^{-\left(\frac{\alpha }{2}+1\right)}\right\}$$ (14)

Where μ is infinitesimal shear modulus, with the constant material parameter b, J_m_ and α are. The fitting of these three models given by Equations (10), (12) and (14) was performed in MATLAB, and the quality of fit for each model was estimated based on the coefficient of determination, R^2^. Significance was considered when the p-value lower than 0.05.

## Results

### Rate-dependent mechanical properties of the infant cerebellum tissue

**Fig 2:**
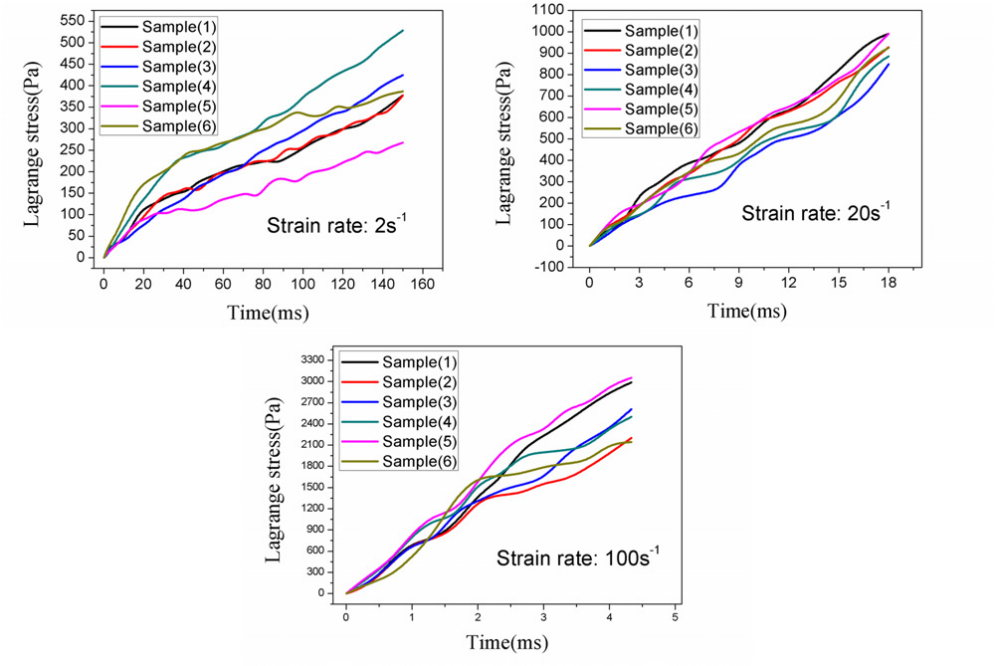
Repeatability of measurements of infant porcine cerebellum tissue up to 30% strain at loading velocity of 10 mms-1 (strain rate 2s-1),100 mms-1 (strain rate 20 s-1), and 500 mms-1 (strain rate100 s-1).

**Fig 3:**
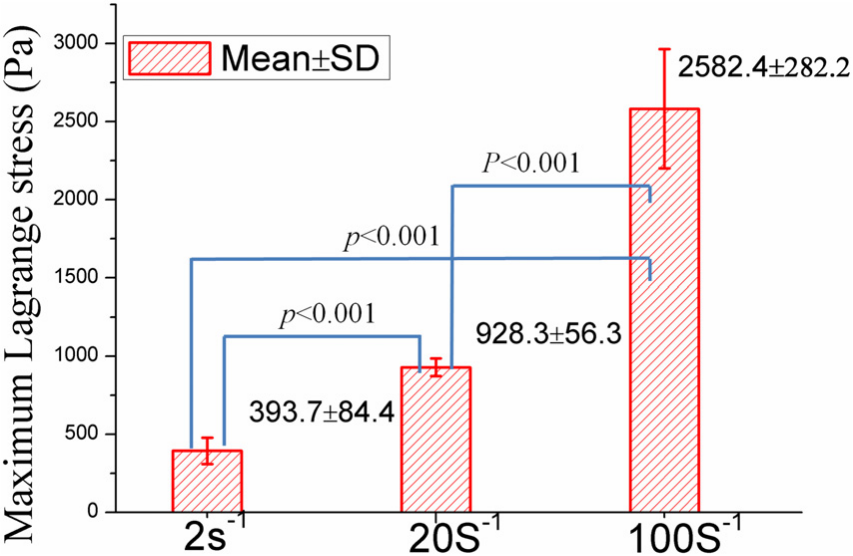
There is a significant difference between maximum Lagrange stress (Mean±SD) of each two loading velocity.

**Fig 4:**
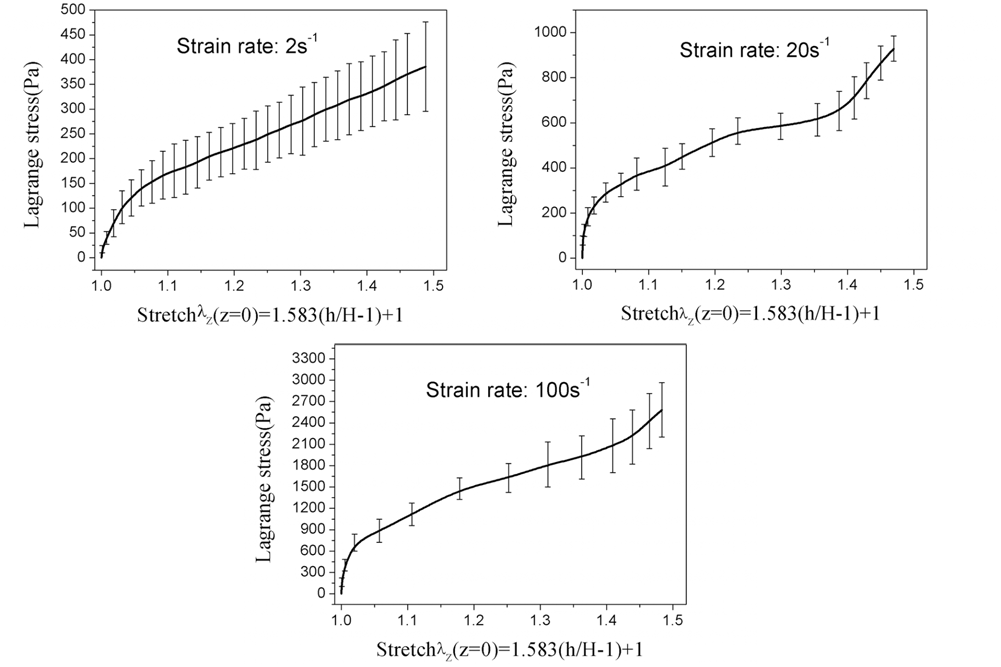
Lagrange stress versus stretch in the plane of symmetry for loading velocity of 10 mms-1 (strain rate 2s-1), 100 mms-1 (strain rate 20s-1), and 500 mms-1 (strain rate100s-1).

To analyze the repeatability of measurements and behavior of tissue at a particular loading velocity, at least six tensile tests for each strain rate of 2^-1^, 20^-1^ and 100s^-1^ up to 30% strain were validly performed. The raw displacement data were processed to have a suitable vertical stretch. The force and displacement data were then converted to average Lagrange stresses (Pa, the vertical force divided by initial cross-sectional area) and stretch ratio λ according to Equation (6). The Lagrange stresses versus time for each strain rate were presented in Fig. 2. The coefficient of variation (standard deviation divided by the mean) for strain rates of 2s^-1^, 20s^-1^, and 100s^-1^ was found to be 0.20–0.42, 0.06–0.25, and 0.10–0.37, respectively. In addition, the Lagrange stresses at each strain rate were statistically different at 30% strain, with (393.7±84.4)Pa, (928.3±56.3)Pa and (2582.4±282.2)Pa, respectively (Fig. 3). It was observed that the infant cerebellum tissue stiffness increased with the loading velocity, indicating a strong stress-strain rate dependence of infant cerebrum tissue (Fig. 4).

The Young’s moduli at each strain rate were calculated from the experimental data shown in Fig. 4. The Young’s moduli E1, E2, and E3 were also obtained as the slope of the stress-strain response in the strain range of 0–0.1 (0–10%), 0.1–0.2 (10–20%), and 0.2–0.3(20–30%) respectively. The estimated Young’s moduli at each strain rate are summarized in Table 1. The data demonstrate significant differences in Young’s modulus determined in the range of 0–10% and 10–20% strain among three different strain rate levels. As for initial Young’s modulus determined in the range of 0–10% strain, no significant differences were found between the strain rate of 2s^-1^ and 20s^-1^. However there is a consistent rise in moduli with increasing strain ranges and with increasing strain rates.

**Table 1:** The Young’s moduli at each strain rate were calculated from the experimental data.

Strain rate	E1(Pa)	E2(Pa)	E3(Pa)
	0–0.1	0.1–0.2	0.2–0.3
2 s^-1^	120.4±69.2	249.1±26.0	339.0±105.4
20s^-1^	246.1±148.6	553.5±37.1	759.3±122.9
100s^-1^	547.6±432.7	1639.6±170.7	2241.6±848.0
Mean	252.2±263.4	588.1±562.0	958.0±822.3

### Fitting of constitutive models

**Fig 5:**
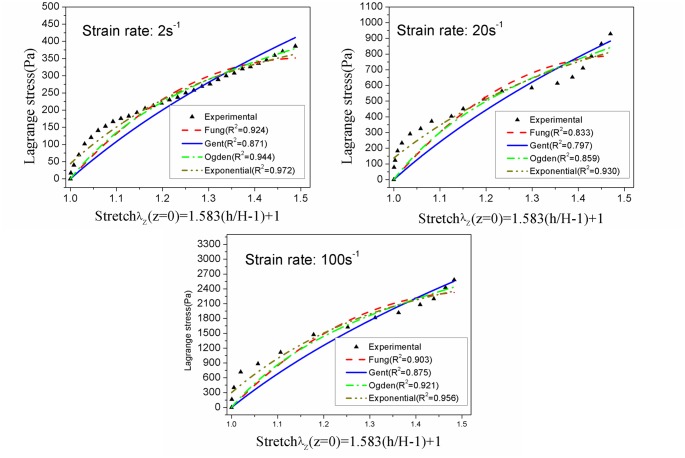
Theoretical curves for the Fung, Gent and Ogden models at each loading velocity.

To well describe the mechanical properties of infant cerebellum tissue, an exponential model has also been applied to observe the tensile deformation at each strain rate. The average Lagrange stress-stretch curves at each loading rate in Fig. 4 were used to fit Fung, Gent, Ogden and exponential models. The infinitesimal shear modulus derived at each strain rate were calculated and showed in Table 2, which demonstrated that the infinitesimal shear modulus increased with loading velocity, but it did not exceed 1500 Pa even at strain rate of 100s^-1^. A good fitting was achieved for Fung model (0.833≤R^2^≤0.924), Gent model (0.797≤R^2^≤0.875) and one-term Ogden model (0.859≤R^2^≤0.944), and the resulting theoretical curves are shown in Fig. 5. In addition, the highest coefficient of determination (0.930≤R^2^≤0.972) was achieved for the Exponential models given by Equations (15), (16) and (17) for each loading velocity.

$$2s^{-1}:\;S_{11}\;=\;-7957.9e^{-\frac{\lambda }{0.33936}}+462.0$$ (15)

$$20s^{-1}:\;S_{11}\;=\;-9882.8e^{-\frac{\lambda }{0.44266}}+1169.6$$ (16)

$$100s^{-1}:\;S_{11}\;=\;-51953.6e^{-\frac{\lambda }{0.33834}}+3003.2$$ (17)

**Table 2:** Mean±SD of infinitesimal shear modulus at each strain rates.

Strain rate	Fung model (Pa)	Gent model (Pa)	Ogden model (Pa)
2 s^-1^	493.0±23.4	396.6±14.3	560.9±14.8
20s^-1^	1198.7±86.4	780.8±80.9	1110.8±176.5
100s^-1^	1259.2±92.9	881.6±74.2	1239.6±132.1

## Discussion and Conclusions

It has been widely accepted that finite-element modeling is a powerful tool to help us understand the mechanism of traumatic brain injury induced by motor vehicle crashes (MVCs). At present, a number of adult human finite-element models have been developed. However, due to paucity of pediatric material property data, the most models of pediatric head used nowadays were derived from the scaled adult head models. So, similar models need to be developed to identify age-specific mechanisms and injury tolerances appropriate for children. This study has presented the results of extension of the infant porcine cerebellum tissue and analyzed mathematical models of the tissue deformation behavior. The maximum coefficient of variation did not exceed 0.5 in this study, indicating a high repeatability of the experiments. In the present study, the infant cerebellum tissue in tension exhibited a strain-rate sensitivity, as is in accordance with the previous researches on the tensile properties of adult brain tissue [9, 10, 11].

One of the practical difficulties in our uniaxial tensile tests of the infant porcine cerebellum tissue was to capture the consistent specimens. Thus, we did not conduct the tensile experiment until the prepared specimen was satisfactory. Data on the viscoelastic properties of human cerebellum measured by Zhang et al [15] using magnetic resonance elastography suggested that the cerebellum was less physically stiff than the cerebrum. The cerebrum tissue properties were age-dependent, with infant tissue being approximately twice as stiff as adult tissue at large deformations, and adult tissue being stiffer than pediatric tissue at very small strains [2, 25]. Until now, no other data existed for infant cerebellum properties in tension and a direct comparison with our research is still impossible. However, the Young’s moduli shown in this study were even no more than 10% of that of adult cerebrum tissue determined by other research groups [10]. And the Lagrange stress of infant cerebellum tissue in our tensile tests did not exceed 3.0kPa, which is less than the results of adult cerebrum at the similar strain rate [11], partly supported by Zhang et al [15]. Another possible explanation for this may lie in the lower myelination and higher water content of the infant cerebellum [26].

According to previous studies [9, 11], many constitutive models proposed to describe the behavior of brain tissue are based on the strain energy function and the isotropic hyper-elastic incompressibility of the brain tissue. Among the Fung, Gent, Ogden, and exponential models, the exponential model is the best to describe the mechanical behavior of infant cerebellum tissue in tension, with excellent agreement between experimental and theoretical results; and the Ogden model is second only to the exponential model. The infinitesimal shear stress derived in Fung, Gent and Ogden models are almost 1/2 less than that of adult cerebrum reported by Rashid et al [11] at the similar strain rates. In addition, due to the significant effects of temperature on brain tissue [13, 27], we conducted all the tensile tests at the consistent room temperature, thus achieving excellent repeatability of measurements. Moreover, as the postmortem time is considered to be the dominant cause of large variations in results [1], all tests were completed within 6h of the infant porcine after sacrifice, which is consistent with previous work [3, 9].

In word, as one of the first steps to characterize the infant cerebellum properties, the results of the study give a new insight into the material properties of brain tissue, which may augment the biofidelity of finite element model of human pediatric cerebellum. However, there still remain certain limitations of this study. First, the main purpose of this study is to determine the strain-rate sensitivity of infant cerebellum. Then, the tests were primarily conducted as a preliminary and tentative research. Second, the specimens used in the experiments contained a mixture of white and gray matter, so these results are mainly useful in modeling the approximate behavior of such composite cerebellum tissue. Third, the study used the *in vitro* experimental environment. Gefen and Margulies [28] suggested that the pressurized vasculature and loss of perfusion pressure have very little effect on the behavior of cerebrum tissue. In fact, the different changes of the infant cerebellum tissue *in vitro* and *in vivo* need to be clarified in the further research.
